# BARI+: A Biometric Based Distributed Key Management Approach for Wireless Body Area Networks

**DOI:** 10.3390/s100403911

**Published:** 2010-04-16

**Authors:** Khaliq-ur-Rahman Raazi Syed Muhammad, Heejo Lee, Sungyoung Lee, Young-Koo Lee

**Affiliations:** 1 Ubiquitous Computing Lab, Department of Computer Engineering, Kyung Hee University (Global Campus), Yongin, Korea; E-Mails: raazi@khu.ac.kr (K.-R.R.S.M.); yklee@khu.ac.kr (Y.-K.L.); 2 Division of Computer & Communication Engineering, Korea University, Seoul 136-713, Korea; E-Mail: heejo@korea.ac.kr

**Keywords:** humanware, healthcare, security, key management, body area networks

## Abstract

Wireless body area networks (WBAN) consist of resource constrained sensing devices just like other wireless sensor networks (WSN). However, they differ from WSN in topology, scale and security requirements. Due to these differences, key management schemes designed for WSN are inefficient and unnecessarily complex when applied to WBAN. Considering the key management issue, WBAN are also different from WPAN because WBAN can use random biometric measurements as keys. We highlight the differences between WSN and WBAN and propose an efficient key management scheme, which makes use of biometrics and is specifically designed for WBAN domain.

## 1. Introduction

### 1.1. Background

Sensor networks are used to monitor chemical, biological, physical, environmental or any other kind of phenomena in real-time environments. Sensor networks consist of resource constrained sensor devices, which relay their sensed data to a central server through the network using wireless communications [1]. This data is processed or used at the central server according to the application requirements. In order to increase efficiency, information is also filtered in the intermediate nodes.

A wireless body area network (WBAN) is formed when sensor nodes are tactfully placed on human body to collect its biometrics or activities. Applications of WBAN include healthcare, lifecare and athlete examination. Healthcare includes care for inpatients especially those who are seriously ill, unconscious or under intensive care. Lifecare includes patients who live their lives normally but may require medical care at any time. For example, lifecare facilities are useful in monitoring health of elderly people and pregnant women in real-time. Lack of timely medical care may cost some people their lives, e.g., heart patients or high risk pregnant women. Also, WBANs are very useful in examining and monitoring an athlete’s body.

The use of WBAN in applications, which are crucial for human life, highlights importance of its security. Apart from making sure that a person’s biometric information is not tampered with, it is important to ensure confidentiality of the person’s information. Key management plays a pivotal role in ensuring data integrity and protecting patient’s private data from eavesdroppers and unauthorized users.

In order to ensure confidentiality and integrity, highly secure state-of-the-art mechanisms such as TLS [2] and Kerberos [3] exist but they are too heavy to run on resource constrained sensor nodes. Mechanisms such as LEAP+ [4], SHELL [5] and MUQAMI+ [6] are resource efficient for sensor nodes but they are designed keeping in mind unattended large scale Wireless Sensor Networks (WSN), in which all nodes may not be in communication range of each other. Apart from being small scale wireless network that can have human intervention, WBAN have all nodes in communication range of each other. Also, application characteristics of WBAN can be exploited to further reduce key management overhead as discussed in [7]. Differences between WSN and WBAN are discussed in detail in the following section (Section 1.2.).

### 1.2. Motivation and Problem Statement

WBAN are adhoc networks formed by sensor nodes placed on different parts of a human body. Sensor nodes have less memory, computation and communication capabilities. Also, they have limited energy resources. Based on above properties, WBAN are classified into same category as WSN and treated in the same way when designing key management schemes. However, we find that WBAN are different from usual WSN in many ways.

Firstly, WBAN and WSN differ in scale. For WSN, number of nodes may be in thousands while WBAN consists of very few nodes, which may not exceed twenty. Obvious reason for this difference is usability. In humanware applications, sensor devices can be placed in watches, lockets or other wearable things. People may not agree to wear a lot of devices. If they do, it hampers their daily routine.

Secondly, nodes in WBAN are very close to each other as opposed to WSN. Nodes in WSN are scattered in large area like battlefield while nodes in WBAN are placed in small area, *i.e.*, a human body. Placing sensor nodes on a human body brings many of them in communication range of each other. Communication protocols have been designed keeping in mind such topology [8].

Thirdly, a compromised node can be physically removed in WBAN, which may not be the case in WSN because human intervention is not always possible in WSN. In WBAN applications, which are crucial for human life, it is essential to physically replace compromised nodes. For example, if there is only one node measuring a serious patient’s heart rate, it must be replaced immediately. Since it is possible to physically remove a compromised node in WBAN, it is not efficient to use node eviction strategies in key management scheme.

Lastly, WBAN are used to measure biometrics from human body. Biometric values exhibit sufficient randomness properties and can be used to generate random numbers for cryptographic keys [9]. [9] uses “the last digit fluctuation method” to generate random sequence from biometric data and extracts the least significant bit from every reading. Also, [9] proves that the least significant bit from every reading have sufficient randomness. According to [9], about *n* readings are required to generate an *n* bit key, which is viable because sensor nodes sense biometrics a lot more often than they relay its values to the central server. There are two reasons for preferring physiological value based keying over pseudo-random number generators: Firstly, pseudo-random number generators require heavy computations as compared to physiological value based keying. Secondly, all random numbers are independent of each other in physiological value based keying as opposed to pseudo-random number generators. In pseudo-random number generators, a mathematical algorithm and a seed value are used to generate random numbers. If the algorithm and the seed value are exposed, the sequence of random numbers becomes deterministic. Also, obtaining truly random seed value is also a challenge. Phenomena that are measured in WSN applications may not have such randomness properties. WBAN can not be treated as a Wireless Personal Area Network (WPAN) because of the same reason. Some researchers use biometrics for key generation [10, 11]. Some of them argue that sensor nodes do not even need to exchange keys [12–14]. They rely on the assumption that two nodes can sense a biometric at the same time. After that, they apply error-correcting codes at both the communicating nodes. Apart from extra computations and time synchronization issues, this assumption imposes another constraint on the network, *i.e.*, some nodes should be able to sense more than one biometric, which may not be practically possible. Also, such schemes do not take into account those nodes, which are not used for sensing biometrics. For more detail, refer to the system model of WBANs described in Section 3.

Differences between WBAN and WSN are summarized in Table 1. The only difference in security requirement of WBAN and WSN evident from Table 1 is that a compromised node in WBAN scenario need not be evicted through software because human intervention is always possible. However, there is also difference between types of attack that can take place through compromised nodes in WBAN and WSN scenarios. In WBAN, we do not need to take care of routing attacks such as selective forwarding, wormhole and sinkhole attacks because many nodes have the cluster head node in their communication range. Nodes, which have very limited communication range, can communicate through one intermediate node. Moreover, due to the fact that WBAN are small scale networks, in which many nodes are in communication range of each other, we do not need to employ strategies to prevent attack propagation in WBAN. Table 2 outlines the differences between security requirements of WBAN and WSN.

From Table 2, it is clear that the security requirements of WBAN are less complex than that of WSN. Also, from Table 1 we learn that we can achieve more efficiency in key management solutions if we exploit the characteristics of WBAN applications while designing key management scheme for WBANs.

### 1.3. Main Contributions

Key management schemes of WSN prove to be overly complex for WBAN because security requirements of WSN are more complex than that of WBAN. Also, key management schemes designed for WSN can not take advantage of the characteristics of WBAN applications in order to achieve more efficiency. Therefore, we present BARI+ (In the conference version, we named this protocol BARI [15]. Since it is improved and described more elaborately in this journal version, we have named it BARI+), which is a distributed key management scheme that fulfills the security requirements (as stated in Table 2) of WBAN. Apart from fulfilling security requirements of WBANs, BARI+ also exploits application characteristics of WBAN to achieve more efficiency.

BARI+ is a distributed key management scheme, which makes use of key refreshment schedules to distribute key management responsibility among all nodes in a WBAN in a fair manner. All nodes in WBAN are able to take part in key management because nodes need not generate keys themselves. After presenting our scheme, its overhead is analyzed and compared with other state-of-the-art schemes. Apart from analyzing storage and communication overhead, security of our scheme is also analyzed.

Rest of this paper is organized as follows: Section 2. outlines the related work followed by section 3., which states the system model and assumptions. Section 4. presents our scheme. Section 5. analyzes our scheme and compares it with other state-of-the-art key management schemes. Section 6. presents simulation results and then Section 7. concludes the paper. In this paper we use many abbreviations and notations like WBAN for wireless body area networks. Refer to Table 3 for complete list of notations used in this paper.

## 2. Related Work

Due to the fact that WBAN consist of sensor nodes, they have been considered similar to WSNs. Therefore, most of the related work is from WSN paradigm. The most simple key management solutions is to distribute keys to each pair of communicating nodes before the deployment and then use them throughout the network lifetime. Extreme care must be taken during key assignments otherwise it may result in inefficient security. For example, same key should not be assigned to multiple pair of nodes within a certain area. Likewise, there are many other issues in key pre-distribution. Efficient mechanisms, which take care of those issues, also exist [16–20].

Main problem with key pre-distribution is that if we keep on using same keys for longer periods of time, they may come under cryptanalytic attacks. When considering network lifetime, Mica2, which is real world example of a sensor node, is a very good example. At full power, its lifetime is expected to be two weeks [21]. In WBAN, network lifetime may be indefinite because nodes’ batteries can be replaced or recharged. Under such circumstances, periodic key refreshment becomes necessary.

Many schemes, which support key refreshment, have been proposed for WSN. Key management scheme of Riaz *et al.* [22] requires the base station to provide public keys to the communicating nodes. Drawback of Riaz’s scheme is that frequent communication with the cluster head node incurs significant communication overhead. Paek *et al.* [23] base their scheme on regional and virtual groups. LEAP+ [4] is a localized scheme and one of the state-of-the-art solutions for WSN. Common drawback of Paek’s scheme and LEAP+ is their assumption that the network is safe during some initial time period. Also, both these schemes are not designed for a scenario, in which all nodes are in communication range of each other.

[24] and [25] use asymmetric cryptography in WSN using Elliptic Curve Cryptography. Apart from being designed for large scale sensor networks, both of these schemes move the additional burden of public key cryptography to the cluster head node. We argue that such strategies should be avoided because the cluster head nodes also have limited battery and they become a single point of failure in case they are compromised. Another drawback of [25] is that it assumes network safety in some initial time period.

SHELL [5] and MUQAMI+ [6] are lightweight solutions and suit the resource constrained sensor nodes well. They also avoid single points of failure in sensor networks. Both these schemes are based on combinatorics and Exclusion Basis System (EBS) matrix [26]. MUQAMI+ improves performance by distributing the key management responsibilities locally. Also, it makes use of key-chains [27], which are based on Lamport’s one-time passwords [28]. However, both these schemes are designed keeping in mind large scale nature of WSN. When applied to small scale networks, their performances drop considerably. Also, EBS based key management schemes are prone to collusion attacks [29].

All of the above schemes are generally efficient in WSN scenarios but none of them makes use of the characteristics of WBAN applications. Also, their designs are overly complex for WBAN scenarios. Previously, researchers have focused on application characteristics of WBANs but their research has been limited to the usage of biometrics values as keys and authentication codes [10, 11] as already discussed in Subsection 1.2. Importance of the research of [10] and [11] is that they have substantially reduced the computation costs involved in generating keys. Also, some researchers have focused on eradicating the need for key exchange [12–14] assuming that two communicating nodes can sense same biometric at the same time and then apply error-correcting codes to agree on a secret key. Eradicating the need for key exchange eradicates communication costs involved in key management. Apart from time synchronization issues, these schemes add another constraint to the network: they require some sensor nodes to sense more than one biometric. Having multiple sensors in a sensor node increases the cost of sensor node and may not be practical in many WBAN scenarios. Authors in [30] have eradicated time synchronization issues by using photoplethysmogram (PPG) signals for key exchange. To study its efficiency, they have also implemented their scheme in hardware [31]. However, issue of multiple sensing still remains a challenge. We propose a complete key management architecture, keeping in mind application characteristics and security requirements of WBAN. Also, our scheme does not have time synchronization and multiple sensing issues. To our knowledge, this is the first key management scheme, which is designed for WBAN and does not have time synchronization and multiple sensing issues.

## 3. System Model and Assumptions

Scenario of WBAN is such that there are few sensor devices, which are capable of measuring biometrics related to human body. These devices are tactically placed on a human body in such a way that they do not hamper daily routine of the human being. Also, there is a Personal Server (PS), which can be a laptop or a hand held device. The PS and all the sensor nodes form a wireless body area network (WBAN). We assume that the PS is pre-loaded with node identities and the sensor nodes are pre-loaded with relevant keys before deployment. For critical scenarios, sensors that are targeted for the same WBAN and the associated PS can be grouped together in advance. Sensor nodes measure biometrics and forward them to the PS. In turn, the PS relays this information to a central server, which we call Medical Server (MS), through the internet.

**Figure 1.**
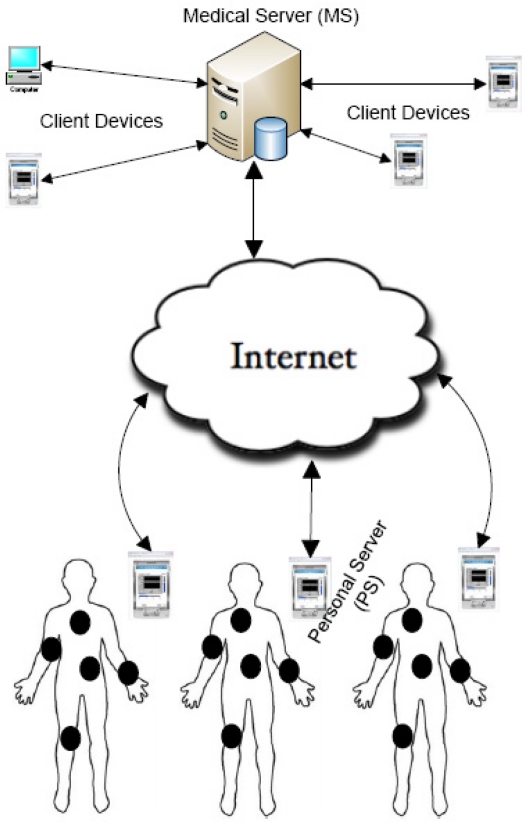
System architecture of wireless body area networks.

Each WBAN is associated with only one body. Multiple WBANs are associated with one central MS. The MS stores and processes information of all the WBANs that are associated with it. An application software running on the MS generates alerts based on the information stored on the server. Also, authorized people can access the required information from the MS. System architecture, as per our assumptions, of WBAN is shown in Figure 1.

We assume that the PS and all sensor devices are constrained in energy because they use rechargeable batteries. Also, we assume that a number of nodes in the network have internal clocks. Unlike other WSN, physical node capture is unlikely to happen in WBAN because all nodes are under human observation. However, node compromise can not be ruled out completely.

## 4. BARI+

**Figure 2.**
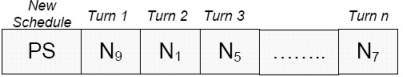
Example of a key management schedule with *n* slots.

Our scheme supports use of biometric measurements as symmetric keys because they posses randomness properties and can be used to generate symmetric keys in WBAN scenarios. This has already been discussed in previous sections. Our scheme makes use of key refreshment schedule, which depicts the turn of each node for key refreshment. The personal server (PS) issues new key refreshment schedule periodically. Each node refreshes the key in the slot allotted to it. The PS can exempt some nodes from their key management responsibilities depending upon their energy level and transmission capabilities. Example of a key refreshment schedule is shown in Figure 2.

Our scheme uses four types of keys to manage a WBAN: communication key, administrative key, basic key and a secret key shared between sensor node and the medical server. Communication key *K_comm_* is a network wide key and is used to transfer data through the network in a secure manner. In our scheme, *K_comm_* is managed by the PS itself. Since *K_comm_* is used very frequently, it may come under cryptanalytic attacks and must be refreshed regularly.

**Figure 3.**
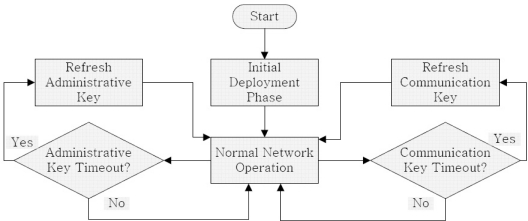
Flowchart of our proposed scheme.

Administrative key *K_admin_* is used to refresh *K_comm_*. *K_admin_* is also a group key but it is not used as frequently as *K_comm_*. Naturally, *K_admin_* is less exposed as compared to *K_comm_*. Although PS is more capable than a sensor node, PS is also a battery powered device. Also, sensor nodes need not generate keys in order to refresh them. Therefore, we use refreshment schedules to distribute the responsibility of key management evenly throughout the network. In order to increase resilience in a WBAN, we can increase the number of administrative keys being used. Figure 3 shows the manner, in which our scheme manages the keys of a WBAN.

In WBAN applications, it is almost impossible for an adversary to compromise a node physically because of human presence. Although it is possible, it is less likely that an adversary can place a malicious node nearby to hack into a node’s system software. Even if such an event occurs, it is a lot easier to detect as compared to WSN because the PS can directly monitor the activity of a compromised node and the compromised can be removed through human intervention. Despite the fact that there are lesser chances of malicious activities in WBAN, it is important to cover all possibilities. Also, *K_admin_* needs to be refreshed through some other key at some point in time. Therefore, we employ basic keys *K_bsc_* in our key management framework. Every node has its own *K_bsc_*, which it shares only with the PS. Key *K_SN_*_,_*_MS_* is a rarely used backup key shared between sensor node and the medical server. *K_SN_*_,_*_MS_* is important and is essential to recover from the compromise of PS or *K_bsc_*.

### 4.1. Initial Deployment

PS is deployed in the beginning. Throughout network lifetime, PS is connected with the medical server through an external secure communication channel, which may be the internet. PS comes pre-loaded with *K_admin_*, *K_comm_* and basic keys of all nodes that are to be deployed in the network. Also, identities and authentication codes of all nodes are pre-loaded in the PS. These codes are used to authenticate the sensor nodes. After the PS is deployed, sensor devices are deployed on various parts of the body. Sensor nodes come pre-loaded with authentication codes of all nodes in the network, *K_admin_* and their respective *K_bsc_* and *K_SN_*_,_*_MS_*. Soon after deployment, every node sends discovery message to the PS as follows:

$$m1:\forall i\;\;\;\;\;\;\;\;\;\mathrm{if}\;\exists \;{\mathrm{SN}}^{i}:{\mathrm{SN}}^{i}\to \mathrm{PS}:E_{K_{\mathrm{admin}}}\{{\mathrm{ID}}^{i}|\mathrm{Auth}\_{\mathrm{Code}}^{i}\}$$

In WBAN, some sensor nodes may have very little communication range. MS informs the PS about deployment of such nodes in advance. If such nodes are to be deployed, the PS commands other nodes to forward discovery messages of such nodes to the PS. After all the sensor nodes are deployed, the PS generates a key refreshment schedule for *K_admin_*. It then broadcasts the refreshment schedule and initial value of *K_comm_* in the network as follows:

$$m2:\mathrm{PS}\to *:E_{K_{\mathrm{admin}}}\{K_{\mathrm{comm}}|\mathrm{Key}\_\mathrm{Ref}\_\mathrm{Schedule}|\mathrm{Auth}\_{\mathrm{Code}}^{\mathrm{PS}}|\mathrm{Timestamp}\}$$

In order to prevent the PS from waiting forever, there is a timer. As soon as the last expected node’s discovery message is received or the timer expires, the PS calculates the refreshment schedule and broadcasts its initial message *m*2. All subsequent nodes are treated as added nodes and deployed through the procedure explained in Subsection 4.3.

### 4.2. Re-keying

In order to refresh *K_comm_*, PS computes a value from biometrics as the value of new *K_comm_*. It then encrypts the new value of *K_comm_* with *K_admin_* and broadcasts it into the network as follows:

$$m1:\mathrm{PS}\to *:E_{K_{\mathrm{admin}}}\{K_{\mathrm{comm}}|\mathrm{Auth}\_{\mathrm{Code}}^{\mathrm{PS}}\}$$

Administrative key is refreshed periodically. When the turn of sensor node *i* arrives, sensor node *i* waits for a certain period of time, computes a new value for *K_admin_* from biometrics and broadcasts it in the network as follows:

$$m1:{\mathrm{SN}}^{i}\to *:E_{K_{\mathrm{admin}}^{\mathrm{old}}}\{K_{\mathrm{admin}}^{\mathrm{new}}|\mathrm{Auth}\_{\mathrm{Code}}^{i}\}$$

When the key refreshment schedule expires, the PS calculates the new schedule, encrypts it in the current value of *K_admin_* and broadcasts it into the network as follows:

$$m1:\mathrm{PS}\to *:E_{K_{\mathrm{admin}}}\{\mathrm{Key}\_\mathrm{Ref}\_\mathrm{Schedule}|\mathrm{Auth}\_{\mathrm{Code}}^{\mathrm{PS}}|\mathrm{Timestamp}\}$$

When a network is deployed, key refreshment timeout of every sensor node is decided according to pre-defined criteria. However, PS can decide to refresh *K_admin_* at any point in time if it detects malicious activities. In such scenario, the PS sends key refresh message to the node, which is supposed to refresh *K_admin_* next time. For example, if it is the turn of sensor node *i* to refresh the administrative key, following messages will be exchanged to refresh *K_admin_*:

$$\begin{array}{l} m1:\mathrm{PS}\to {\mathrm{SN}}^{i}:E_{K_{\mathrm{admin}}}\{\mathrm{Key}\_\mathrm{Refersh}\_\mathrm{Msg}|\mathrm{Auth}\_{\mathrm{Code}}^{\mathrm{PS}}|\mathrm{Timestamp}\}\\ m2:{\mathrm{SN}}^{i}\to *:E_{K_{\mathrm{admin}}^{\mathrm{old}}}\{K_{\mathrm{admin}}^{\mathrm{new}}|\mathrm{Auth}\_{\mathrm{Code}}^{i}\} \end{array}$$

In order to maintain forward secrecy, *K_admin_* needs to be refreshed through *K_bsc_* regularly. Also, *K_admin_* needs to be refreshed through *K_bsc_* in case of sensor node compromise. In order to refresh *K_admin_* through *K_bsc_*, the PS computes new values of basic keys and refreshes *K_admin_* using *K_bsc_* of the sensor nodes:

$$\begin{array}{l} m1:\forall i\;\;\;\;\;\;\;\;\;\mathrm{if}\;\exists \;{\mathrm{SN}}^{i}:\mathrm{PS}\to {\mathrm{SN}}^{i}:E_{K_{\mathrm{bsc}\_\mathrm{old}}^{i}}\{K_{\mathrm{admin}}|K_{\mathrm{bsc}\_\mathrm{new}}^{i}|\mathrm{Auth}\_{\mathrm{Code}}_{\mathrm{new}}^{i}|\mathrm{Auth}\_{\mathrm{Code}}^{\mathrm{PS}}\}\\ m2:\mathrm{PS}\to *:E_{K_{\mathrm{admin}}}\{K_{\mathrm{comm}}|\mathrm{Auth}\_{\mathrm{Code}}^{\mathrm{PS}}\} \end{array}$$

Although basic keys are used only once and refreshed after every use, it is possible that they need to be refreshed using some other key. For example, if the PS is compromised. Therefore, we think it is important to have a procedure to recover from such catastrophic failures. In such scenario, authentication code of the PS is also refreshed. If a new PS is deployed, it comes pre-loaded with *K_admin_* and *K_comm_*. MS sends identities, authentication codes and basic keys of the sensor nodes to the newly deployed PS. If the PS is not replaced, MS sends new values of *K_bsc_* to the PS. Also, MS encrypts new values of *K_bsc_*, along with the new authentication code of PS, in *K_SN_*_,_*_MS_* of all sensor nodes and sends them to the PS through an external secure communication channel, which may be the internet. After receiving messages encrypted in *K_SN_*_,_*_MS_* of the sensor nodes, PS just forwards them to the respective sensor nodes. Whenever *K_bsc_* is refreshed, *K_admin_* and *K_comm_* are also refreshed. Following message exchanges take place between the PS and sensor nodes when *K_bsc_* is refreshed using *K_SN_*_,_*_MS_*:

$$\begin{array}{l} m1:\forall i\;\;\;\;\;\;\;\;\;\mathrm{if}\;\exists \;{\mathrm{SN}}^{i}:\mathrm{PS}\to {\mathrm{SN}}^{i}:E_{K_{\mathrm{SN},\mathrm{MS}\_\mathrm{old}}^{i}}\{K_{\mathrm{bsc}}^{i}|\mathrm{Auth}\_{\mathrm{Code}}_{\mathrm{new}}^{\mathrm{PS}}|K_{\mathrm{SN},\mathrm{MS}\_\mathrm{new}}^{i}|\mathrm{Auth}\_{\mathrm{Code}}^{\mathrm{MS}}\}\\ m2:\forall i\;\;\;\;\;\;\;\;\;\mathrm{if}\;\exists \;{\mathrm{SN}}^{i}:\mathrm{PS}\to {\mathrm{SN}}^{i}:E_{K_{\mathrm{bsc}\_\mathrm{old}}^{i}}\{K_{\mathrm{admin}}|K_{\mathrm{bsc}\_\mathrm{new}}^{i}|\mathrm{Auth}\_{\mathrm{Code}}_{\mathrm{new}}^{i}|\mathrm{Auth}\_{\mathrm{Code}}^{\mathrm{PS}}\}\\ m3:\mathrm{PS}\to *:E_{K_{\mathrm{admin}}}\{K_{\mathrm{comm}}|\mathrm{Auth}\_{\mathrm{Code}}^{\mathrm{PS}}\} \end{array}$$

Note that *K_SN_*_,_*_MS_* is refreshed whenever it is used. Also, the PS does not get to know key *K_SN_*_,_*_MS_* of any sensor node. Remaining key refreshment schedule is followed after the refreshment of *K_admin_* irrespective of the way *K_admin_* is refreshed.

### 4.3. Node Addition

In some cases, new nodes are added to the network or the existing nodes are replaced. One possible scenario of node addition can be the deployment of a new device to monitor some biometric. Similarly, one possible scenario of node replacement is malfunction of a device. Under such circumstances new nodes are added to the network.

If new nodes are to be added in the network, MS informs PS about new deployments by sending identities, basic keys and authentication codes of new nodes to the PS. Also, MS informs the PS about initial value of *K_admin_* that is preloaded into the new nodes. All this communication is done through an external secure communication channel. Under normal circumstances, if the PS receives messages from stranger nodes, it ignores them and indicates malicious activity on its own output. If informed by the MS, the PS expects discovery messages from new nodes. This is important because otherwise malicious nodes can drain its energy by sending fake discovery messages. New nodes send their respective discovery messages encrypted in the pre-loaded value of *K_admin_* as follows:

$$m1:\forall {\mathrm{SN}}^{j}\in \{\mathrm{New}\_\mathrm{Nodes}\}:{\mathrm{SN}}^{j}\to \mathrm{PS}:E_{K_{\mathrm{admin}}^{\mathrm{pre}-\mathrm{load}}}\{{\mathrm{ID}}^{j}|\mathrm{Auth}\_{\mathrm{Code}}^{j}\}$$

If nodes, which have very limited communication range, are deployed, then the PS commands other sensor nodes to forward their discovery messages to the PS. PS waits for all expected nodes for a certain period of time. After that, it broadcasts the remaining key refreshment schedule and current values of *K_comm_* and *K_admin_* to newly deployed nodes as follows:

$$m2:\mathrm{PS}\to *:E_{K_{\mathrm{admin}}^{\mathrm{pre}-\mathrm{load}}}\{K_{\mathrm{comm}}|K_{\mathrm{admin}}|\mathrm{Remaining}\_\mathrm{Sched}|\mathrm{Auth}\_{\mathrm{Code}}^{\mathrm{PS}}|\mathrm{Timestamp}\}$$

All nodes, except the newly deployed ones, ignore such message from the PS. Newly deployed nodes can participate in key refreshment procedure after the next key refreshment schedule is issued by the PS.

## 5. Analysis and Comparison

In this section, we establish our claims regarding efficiency of our scheme BARI+ by analyzing its storage and communication overheads and comparing it with other key management schemes. Also, security analysis of our scheme is presented at the end of this section. According to our knowledge, this is the first key management scheme that is proposed for WBAN and does not require multiple sensing. Therefore, we compare our scheme with two other state-of-the-art key management schemes, which are designed for WSN, LEAP+ [4] and MUQAMI+ [6]. SHELL [5] is also a state-of-the-art key management scheme for WSN but it is not applicable to WBAN because it requires services of neighbouring cluster head nodes, which may not be present in WBAN scenario. When applying LEAP+ and MUQAMI+ in WBAN scenario, we assume that the PS acts as cluster head node and all nodes on one body are part of the same cluster. Also, cluster can not span multiple bodies.

### 5.1. Storage Overhead

Storage and exchange of authentication codes is common in all key management schemes. Also, storage requirements of authentication codes do not make much difference when key management schemes are compared with respect to their storage overhead. For simplicity, storage requirements of authentication codes is not included in storage analysis. Considering storage overhead of sensor nodes, only four keys are stored: *K_comm_*, *K_admin_*, *K_bsc_* and *K_SN_*_,_*_MS_*. Apart from that, key refreshment schedule is stored on sensor nodes. A sensor node keeps track of its turn with the help of two short integers. One integer contains a counter to keep track of its turn. The other one indicates timeout after which it refreshes *K_admin_*. If we consider that a short integer requires 2 bytes and key length is *z* bytes, Then the storage requirement of a sensor nodes becomes:

(1) $${\mathrm{SR}}_{\mathrm{SN}}^{\mathrm{BARI}+}=(4\times z)+4$$

PS stores *K_bsc_* of all sensor nodes, *K_admin_* and *K_comm_*. Also, it stores complete key refreshment schedule for *K_admin_*. Storing a sensor node’s identity requires 2 bytes. Another 2 bytes are required to specify timeout after which sensor node refreshes *K_admin_*. So, the storage requirements of PS becomes:

(2) $${\mathrm{SR}}_{\mathrm{PS}}^{\mathrm{BARI}+}=((r+2)\times z)+(4\times r)$$

where *r* is the number of nodes in WBAN formed on a body. Note that key *K_SN_*_,_*_MS_* is not stored on the PS.

Storage requirements of a node (sensor node or personal server) in LEAP+ is fairly straightforward. Apart from pairwise keys shared with each node in its cluster, every node stores its cluster key and the communication key. So, the storage requirement of a node in LEAP+ becomes:

(3) $${\mathrm{SR}}_{\mathrm{PS}\vee \mathrm{SN}}^{\mathrm{LEAP}+}=z\times (r+2)$$

In MUQAMI+, each PS node has to store *K_comm_* and *K_cn_*_,_*_ch_*. Also, PS has to store *K_ch_*_,_*_sn_* of all SN nodes and key-chains of key *K_ch_*_,_*_kg_* of all KG nodes in its cluster. In addition to that, PS has to store EBS matrix. If EBS data for each node takes 4 bytes (2 bytes for storing node identity and 2 bytes for storing key pattern), it takes 4 × *r* bytes to store EBS matrix. So, the average storage requirement of a PS node (in bytes) of MUQAMI+ becomes:

(4) $${\mathrm{SR}}_{\mathrm{PS}}^{\mathrm{MUQAMI}+}=(z\times ((l\times (k+m))+r-(k+m)+2))+(4\times r)$$

where *l* is the length of key-chains [27], which are used by MUQAMI+ for key management and *k* and *m* are EBS [26] parameters. In MUQAMI+, SN nodes have to store *k* admin keys apart from four other keys: *K_ch_*_,_*_sn_*, *K_comm_*, *K_bsc_* and *K_disc_*. So, the average storage requirement of a sensor node in MUQAMI+ can be expressed as:

(5) $${\mathrm{SR}}_{\mathrm{SN}}^{\mathrm{MUQAMI}+}=z\times (k+4)$$

Among sensor nodes, MUQAMI+ also has key generating (KG) nodes, which store two key-chains: one for the admin key, which it generates and one for *K_ch_*_,_*_kg_*. Also, KG nodes store *k* − 1 EBS keys along with three other keys: *K_comm_*, *K_bsc_* and *K_disc_*. So, the average storage requirement of a KG node can be expressed as:

(6) $$\begin{array}{lll} {\mathrm{SR}}_{\mathrm{KG}}^{\mathrm{MUQAMI}+} & = & z\times (2\times l+(k-1)+3)\\ & = & z\times (2\times (l+1)+k) \end{array}$$

In MUQAMI+, we have *k* + *m* KG nodes out of a total of *r* nodes in a cluster. Therefore, average storage requirement of each node within a cluster comes out to be:

(7) $$\begin{array}{lll} {\mathrm{SR}}_{\mathrm{SN}\cup \mathrm{KG}}^{\mathrm{MUQAMI}+} & = & z\times \frac{\left(r-(k+m))(k+4)+(k+m)(2(l+1)+k\right)}{r}\\ & = & z\times \frac{r\times (k+4)+(k+m)+(2\times (l+1)-4)}{r}\\ & = & z\times ((k+4)+\frac{2\times (l-1)\times (k+m)}{r}) \end{array}$$

Note that (*k* + *m*) << *r* only for large scale networks. For small scale networks like WBAN, *k* and *m* are comparable to *r*, which degrades the performance of MUQAMI+ considerably.

Table 4 compares the storage requirements of BARI+ with MUQAMI+ and LEAP+. It is clear from Table 4 that storage overhead of our scheme is less as compared to other schemes especially on sensor nodes.

### 5.2. Communication Overhead

Communication is the most energy consuming activity in WBAN. Since all nodes are in communication range of each other, we only need to analyze average number of messages transmitted by each type of node in every phase. Initial deployment phase of BARI+ is very lightweight and simple because every node has to send one message each.

Initial deployment phase of MUQAMI+ is also simple. Every sensor node has to send one discovery message each. In return, the PS has to send one message to each node in the network, which makes the total number of messages transmitted by the PS equal to *r*. In LEAP+’s initial deployment phase, the PS has to send one broadcast message to all nodes in the network. All nodes reply and pair-wise keys are established. After that, it sends its cluster key to each of the *r* nodes one by one and then broadcasts its group key in the network. Also, it replies to the initial messages sent by other nodes. So, the average number of messages transmitted by PS in the initial deployment phase of LEAP+ becomes:

(8) $$\begin{array}{lll} \mathrm{Avg}\_\mathrm{Msg}\_\mathrm{Count}\_{\mathrm{Init}}_{\mathrm{PS}}^{\mathrm{LEAP}+} & = & (2\times r)+2\\ & = & 2\times (r+1) \end{array}$$

The sensor nodes does not have to broadcast the communication key. Therefore, average number of messages transmitted by sensor nodes in the initial deployment phase of LEAP+ becomes:

(9) $$\mathrm{Avg}\_\mathrm{Msg}\_\mathrm{Count}\_{\mathrm{Init}}_{\mathrm{SN}}^{\mathrm{LEAP}+}=(2\times r)+1$$

Comparison of our scheme with MUQAMI+ and LEAP+ is given in Table 5, which indicates that our scheme BARI+ is more efficient as compared to other schemes. Nodes are added in the same way as they are initially deployed.

In BARI+, PS broadcasts one message to refresh communication key. Similarly, PS broadcasts one message in the network to refresh communication keyin LEAP+ too. Both in BARI+ and LEAP+, sensor nodes need not send any message to refresh communication key. In MUQAMI+, PS sends *k* + *m* messages to the key generating nodes, which in turn broadcast one message each. So, average number of messages transmitted by a sensor node for communication key refreshment is expressed as:

(10) $$\mathrm{Avg}\_\mathrm{Msg}\_\mathrm{Count}\_\mathrm{Rekey}\_{\mathrm{Comm}}_{\mathrm{SN}}^{\mathrm{MUQAMI}+}=\frac{k+m}{r}$$

Comparison of communication overhead for refreshment of communication keyis given in Table 6.

Refreshment of administrative key is also lightweight in our scheme. In order to refresh administrative key, each node sends one message in every schedule. If all nodes participate in administrative key refreshment, average number of messages sent by each node to refresh *K_admin_* one time comes out to be 1/*r*. However, *K_admin_* is also refreshed by *K_bsc_*. If refreshed through *K_bsc_*, PS sends two messages for the purpose. If *K_admin_* is refreshed by *K_bsc_ y* times in every key refreshment schedule, then average number of messages transmitted by PS for administrative key refreshment becomes:

(11) $$\mathrm{Avg}\_\mathrm{Msg}\_\mathrm{Count}\_\mathrm{Rekey}\_{\mathrm{Admin}}_{\mathrm{PS}}^{\mathrm{BARI}+}=\frac{(2\times y)+1}{r}$$

In LEAP+, every node has to send one message to each of *r* other nodes in the network. In MUQAMI+, PS has to send *k* + *m* messages to key generating nodes and one message after every *l* key refreshments to get new seed values for key-chains. So, average number of messages transmitted by PS for refreshment of administrative key in MUQAMI+ becomes:

(12) $$\mathrm{Avg}\_\mathrm{Msg}\_\mathrm{Count}\_\mathrm{Rekey}\_{\mathrm{Admin}}_{\mathrm{PS}}^{\mathrm{MUQAMI}+}=(k+m)\times (1+(1/l))$$

Similarly, average number of messages transmitted by a sensor node for refreshment of administrative key in MUQAMI+ comes out to be:

(13) $$\mathrm{Avg}\_\mathrm{Msg}\_\mathrm{Count}\_\mathrm{Rekey}\_{\mathrm{Admin}}_{\mathrm{SN}}^{\mathrm{MUQAMI}+}=\frac{\left(k+m\right)}{r}\times (1+(1/l))$$

Table 7 compares our scheme BARI+ with MUQAMI+ and LEAP+ in administrative key refreshment phase.

### 5.3. Security Analysis

Establishing efficacy of a key management scheme for WBAN in different attack scenarios is as important as establishing its energy efficiency. In fact, a key management scheme is useless if it does not fulfill security requirements of the target network. This section analyzes security of our scheme from different perspectives. Also, analysis of protection against various attacks, applicable to WBAN domain, is included in this section. We refer to [32] for the list of attacks that can take place is WSN.

#### 5.3.1. Authentication

In order to provide authentication, our scheme uses authentication codes in all communications. Also, it provides mechanisms to refresh them. In this way, all receiving nodes know origins of a message. If a message does not have a valid authentication code, it is discarded and malicious activity is indicated. If an illegitimate node sends a message, containing authentication code of a legitimate node, the legitimate node indicates this malicious activity.

#### 5.3.2. Confidentiality

All message exchanges are secured using secret keys. A passive adversary, listening to communications, can not comprehend messages unless it obtains secret keys. However, a passive adversary can carry out cryptanalytic attacks on secret keys. To avoid cryptanalytic attacks, keys are refreshed at regular intervals.

#### 5.3.3. Forward Secrecy

It is important to maintain forward secrecy during key refreshment. In our scheme, *K_comm_* is used for all data communication whereas only purpose of *K_admin_* is to refresh *K_comm_*. Therefore, we use *K_admin_* to refresh itself for certain time period, which depends on required security level. After that, we use *K_bsc_* to refresh *K_admin_*. *K_bsc_* is refreshed whenever it is used. Although, this does not provide complete forward secrecy, it does mitigate the problem to an acceptable level. Also, same level of forward secrecy is achieved by those key management schemes, with which our scheme is compared in this section.

#### 5.3.4. Replay Attacks

In replay attack, an adversary listens to communication, stores messages in its memory and transmits them again at a later time. For messages that are vulnerable to replay attack, our scheme makes use of timestamps. If a packet is replayed, it is ignored and malicious activity is in indicated. Timestamps are not used in all messages. For example, timestamps are not required for key refresh messages. If refresh message for *K_comm_* is replayed, it only results in indication of malicious activity. If refresh message of *K_admin_* is replayed, it does not have any meaning because *K_admin_* has already been refreshed. It also results in indication of malicious activity.

#### 5.3.5. Node Compromise

Probability of node compromise is less in WBAN scenario as compared to WSN scenario. Despite that, key management scheme for WBAN must be able to guard against node compromise. In our scheme, PS uses *K_bsc_* to send new values of *K_admin_* to all sensor nodes except the compromised ones. After that, *K_admin_* is used to refresh *K_comm_*. If PS is compromised, new PS is deployed and *K_SN_*_,_*_MS_* is used to verify the new PS and refresh *K_bsc_*. After that, *K_admin_* and *K_comm_* are refreshed subsequently.

#### 5.3.6. Routing Attacks

In WBAN, PS is in direct communication range of many nodes. Nodes, which have very limited communication range, normally select one nearby node to relay their information to the PS. Therefore, WBAN is not much vulnerable to routing attacks such as *selective forwarding*, *sinkhole*, *sybil*, *wormhole* and attacks, in which routing information is spoofed, altered or replayed.

#### 5.3.7. Other Attacks

Other attacks include *flooding*, *desynchronization*, *hello flood* and *acknowledgement spoofing*. In *flooding*, an outsider node tries to carry out denial-of-service by establishing excessive useless connections with a node. In *desynchronization*, adversary tries to disrupt normal communication by repeated spoofing. In *hello flood* attack, adversary uses a high power radio transmitter to make every node believe that the adversary is its neighbour. It then floods the network with hello packets. In *acknowledgement spoofing*, adversary tries to spoof acknowledgement of a packet, which it overhears. Our scheme provides adequate protection against all these attacks because nodes in our scheme ignore all communication from stranger nodes except during initial deployment phase and node addition phase. Reliable authentication mechanism prevents outsider attacks in these two phases too.

## 6. Simulation Results

In our simulations, assumed network architecture was similar to the one shown in Figure 1. Our scheme BARI+ is compared with two other schemes MUQAMI+ [6] and LEAP+[4], which are state-of-the-art key management schemes for WSNs. MUQAMI+ uses EBS matrices and key-chains. EBS parameters *k* and *m* were assumed to be *k* = *m* = 4, so that ample key combinations are left for addition and replacement of nodes in the network. Also, key-chain length in MUQAMI+ was assumed to be 32 so that both storage and communication costs can be kept within practical limits. Number of sensor nodes was assumed to be 15 and key size was assumed to be 16 bytes in our simulations. Moreover, it was assumed that in BARI+, *K_admin_* is refreshed through *K_bsc_* every time a key refreshment schedule expires. Simulations were programmed in “Tools Command Language (tcl8.0)”, which is used to program ns-2 simulations.

Our scheme uses biometrics as keys and need not generate them but other schemes were not designed to take advantage of this property of WBANs. Therefore, cost of key generation is also included in our simulations. [33] states that an 8 MHz processor like ATMEGA128L CPU can generate 50,000 random bytes per minute. According to [33], generating a key or a seed value takes 19.2 ms on 8 MHz processor.

According to G. Xing *et al*. [34], range of data transmission of a sensor node is between −20 dBm and 10 dBm. In WBAN scenario, all nodes are nearby and the ones, participating in key management, are in communication range of each other. Therefore, only one power level was assumed for message transmission. In our simulations, transmission power level was assumed to be 0 dBm (1 mW). Power level during reception and computation phases was assumed to be −10 dBm (0.1 mW). Power level for computation phase was included because computation costs were included considered in our simulations. Usage of MICA2 motes, which have ATMEGA128L CPU as mentioned in [21], was assumed. Moreover, usage of SHA1 hashing scheme and RC5 cipher algorithm was assumed. According to [35], hashing for 16 bytes using SHA1 algorithm takes approximately 3.7 ms; both encryption and decryption for the same length of data using RC5 algorithm takes approximately 3.25 ms on ATMEGA128L CPU.

Apart from power levels, bandwidth of transmission link needs to be consideration. [21] suggests that the application level bandwidth in WSN is around 19.2 kbps whereas [34] suggests that it is around 6 kbps. In our simulations, application level bandwidth was assumed to be 19.2 kbps. Similar results were found when simulations, assuming application level bandwidth to be 6 kbps, were performed.

With the above set of simulation parameters, average energy consumed by PS and SN nodes during initial deployment phase, administrative key refreshment phase and communication key refreshment phase was recorded. For each phase, our simulations had more than 70 iterations. For MUQAMI+, weighted average of sensor nodes and key generating nodes was recorded as average energy consumed by SN nodes. Weights were set according to the number of number of key generating nodes in a network, *i.e.*, *k* + *m* = 8 in our case. Graphs are plotted on logarithmic scale because of large differences in readings of different schemes.

**Figure 4.**
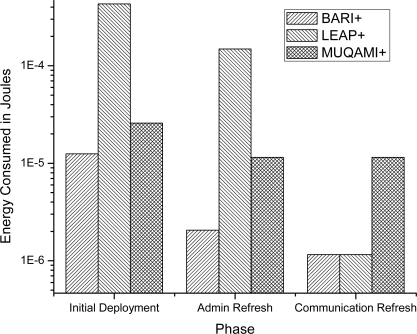
Comparison of Average Energy Consumed by a Sensor Node in different phases of each scheme.

**Figure 5.**
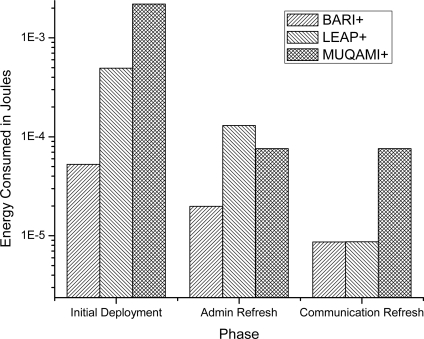
Comparison of average energy consumed by a personal server in different phases of each scheme.

**Figure 6.**
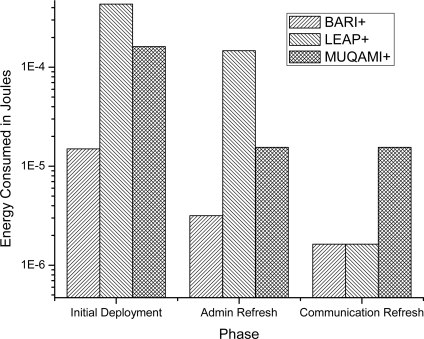
Comparison of average energy consumed by a node (including sensor nodes and the personal server) in different phases of each scheme.

Figure 4 compares the average energy consumed by a sensor node in each of the three schemes in all three phases. Our scheme proves to be more efficient than MUQAMI+ in all the three phases and better than LEAP+ in initial deployment and administrative key refreshment phase. We observe similar results when we compare the average energy consumption of a personal server in each of the three schemes in all three phases in Figure 5. Figure 6 compares the average energy consumed by a node (taking into account sensor nodes and the personal server) in each of the three schemes in all three phases.

## 7. Conclusions and Future Work

This paper highlights differences between WSN and WBAN in terms of application characteristics and security requirements. It establishes that key management protocols for generic applications of WSN are overly complex for WBAN scenario and can not exploit the application characteristics of WBAN. After that, it presents BARI+, which is a key management scheme designed specifically for WBAN applications. Also, it provides analysis of our scheme and its comparison with other schemes.

Apart from attack prevention, it is also important to focus on attack detection in order to provide a complete security solution. Future direction of this research aims to focus on the detection of different types of attacks in WBAN.

## Figures and Tables

**Table 1.** Differences between WBAN and WSN.

	**WBAN**	**WSN**
**Scale**	Small scale (Number of nodes may not exceed 20)	Large scale (Number of nodes may exceed even 1,000)
**Size of Operational Area**	Very small (Size of human body). All nodes may be in communication range of each other	Spans area like battlefields or natural habitat large
**Human Intervention**	Possible rather inevitable in some cases	Not possible in most cases
**Key Management Support from application**	Yes, Sensor nodes need not generate random numbers	No

**Table 2.** Differences between the security requirements of WBAN and WSN.

	**WBAN**	**WSN**

**Message Integrity**	Required	Required
**Node Authentication**	Required	Required
**Prevention from Eavesdropping**	Required	Required
**Node eviction through software**	Not necessary	Required
**Strategies to prevent routing attacks**	Not required	Required
**Prevention of attack propagation**	Not required	Required

**Table 3.** List of Used Notations.

*WSN*	**Wireless Sensor Network**
*WBAN*	**Wireless Body Area Network**
*MS*	**Medical Server**
*PS*	**Personal Server**
*SN^i^*	**Sensor Node i**
$K_{\mathrm{SN},\mathrm{MS}}^{i}$	**Key shared between Node***i***and the MS. It is preloaded in every node and refreshed whenever it is used**
$K_{\mathrm{bsc}}^{i}$	**Basic Key of Node***i***shared with the PS. It is preloaded in every node and is refreshed whenever it is used**
*K_comm_*	**Communication Key**
$K_{\mathrm{admin}}^{i}$	**Administrative Key***i*
*mi*	**Message number***i***in a particular communication sequence**
*E_K_*{*A*|*B*}	**Values A and B are put together in a block/chunk and then the chunk is encrypted using Key***K*

**Table 4.** Storage requirements (in bytes) of each type of node in all three schemes.

	**Personal Server**	**Sensor Node**
**MUQAMI+**	{*z* × {[*l* × (*k* + *m*)] + *r* − (*k* + *m*) + 2}} + (4 × *r*)	(*z* × ((*k* + 4) + [(2 × (*l* − 1) *×* (*k* + *m*))*/r*]))
**LEAP+**	*z* × (*r* + 2)	*z* × (*r* + 2)
**BARI+**	[(*r* + 2) × *z*] + (4 × *r*)	(4 × *z*) + 4

**Table 5.** Average number of messages transmitted by each type of node in initial deployment phase of all three schemes.

	**Personal Server**	**Sensor Node**
**MUQAMI+**	*r*	1
**LEAP+**	2 × (*r* + 1)	2 × *r* + 1
**BARI+**	1	1

**Table 6.** Average number of messages transmitted by each type of node when communication key is refreshed in all three schemes.

	**Personal Server**	**Sensor Node**
**MUQAMI+**	*k* + *m*	(*k* + *m*)*/r*
**LEAP+**	1	−
**BARI+**	1	−

**Table 7.** Average number of messages transmitted by each type of node when administrative key is refreshed in all three schemes.

	**Personal Server**	**Sensor Node**
**MUQAMI+**	(*k* + *m*) × (1 + (1*/l*))	((*k* + *m*)*/r*) × (1 + (1*/l*))
**LEAP+**	*r*	*r*
**BARI+**	((2 × *y*) + 1)*/r*	1*/r*
